# Peer review of "Effects of diversity and coalescence of species assemblages on ecosystem function at the margins of an environmental shift"

_PeerJ, doi:10.7717/peerj.8608_

## Round 0.1 · original submission · Major Revisions

Dear authors,

I have received the comments of the two referees. Both consider that the ms is potentially interesting, but they find important concerns that should be considered; I think that you can make this a much more meaningful paper if you consider their helpful comments (specially concerning the study context, hypothesis, analysis and figures). Please, pay also particular attention to the conclusions you extract from your results and interpretation of results. It is unlikely to pass through review successfully unless you follow all the points provided by the referees. In addition to their comments, please, consider the following minor points:

Introduction
Lines 34-36: “In this experiment…” this is not the good place for that. Please move it at the last paragraph of the introduction section (that beginning in line 49 “In this study…”), or remove it, if it is redundant with information on that paragraph.

Lines 72-75: This sentence doesn’t fit well in the introduction section; it should be moved to the end of the discussion section, for example, as a part of the conclusion.

Methods
Lines 80-81: “0, 5, 9 and 13 psu” information redundant; write it only one time

·

Basic reporting

1. The language used in this manuscript is clear and unambiguous, with profession English used throughout the document
2. The literature cited is generally sufficient, but I think the manuscript would be improved with a slightly more in depth discussion of the effects of salinity on biological communities. For instance, explain how salinity affect communities through direct and indirect impacts would make it easier to follow. In addition, the study is about coastal ponds, but the authors do not explain their importance to the reader. As these unique ecosystems due to their biodiversity? Position in the landscape? Biogeochemical importance in the cycling of a select nutrients? Are they a good model system? This can easily be fixed.
3. The article is structure correctly. However, I think the figures could be improved
a. Figure 4 and 5 – There are a total of 6 PCoA figures, but only a total 2 axis one values and a total of 2 axis 2 values. I would expect to see axis values for each PCoA ( a total of 6 axis 1 values, and a total of 6 axis 2 values). Maybe I am missing something, but I think the authors could clarify this discrepancy.
4. Tables and Figures:
a. The circles and triangles look a bit pixelated, placing boarders around the icons would help, and potentially averaging the treatment groups to one point might help clean the figures up. In addition, I personally like the figures style in in Werba’s master thesis better then these figures ( figures 2-4, regression lines with confidence intervals, no data points). I find the trend much easier to follow, and seeing as you are providing all the raw data any reader could take the data points and plot them. Just a suggestion.
b. Figure 2 - Not sure that running a linear regression is the most meaningful way to interpret these results. If you do decide to keep the regression in, I would think it would be important to report the slopes and assess how different the slopes are from one another – if your goal to perform some type of prediction. However, I could see you still using a GLM and just running an ANOVA on these results. While you wouldn’t get the predictive slope formula, it would highlight the differences between treatment groups, which might be more appropriate for these types of analyses.
c. Figure 7 – are you sure this isn’t a unimodal or “bell curve” response? That would be a very interesting result, suggesting that intermediate levels of microbial richness have the greatest rates of carbon mineralization. You could speculate that at these levels community members have synergistic relationships, but as the richness becomes saturated negative interactions and competitions reduce rates of carbon mineralization.
5. Raw Data
a. The authors have done a great job of providing the raw data from this project via github

Experimental design

1. The research is original to the best of my knowledge, and conforms to PeerJ Aims and Scopes
2. The researchers clearly state they objective in line 49-51, “ i.e. how secondary salinity + mixing fresh and salt taxa affect diversity and composition of zoo. and bac. and downstream ecosystem functions” I wonder if this is really representative of the experiment they performed, or if the authors could craft/ edit this question to be more representative of their 1) study system, 2) study design, and 3) results. I think the first place to start would to be to include the idea of coastal ponds and “overwashing” into your objective, as your experiment is very tailored to this phenomenon. Also, I think your native vs. non native liter decomposition results are very interesting, maybe you would have a main objective and then 2-3 hypotheses that would either support or disagree this objective. I think the authors could make the knowledge gap more apartment by simply relating this to coastal ponds – what is going to happen to these “unique?, diverse?, important?” systems.
3. Methods – methods are pretty thorough, but could use some clarification/ addition information in order to make them replicable.
a. Line 97 – where did you collect these samples from? Water column ( top, middle bottom) did sediments make it into your samples?
b. Line 100-102, peat moss and sand, were these purchased or collected from the environment? Did you sterilize before adding to the microcosm? If you did not, can you address how you think the endemic populations of these substrates would/ would not affect the composition of your biological communities?
c. General – were your mesocosms mixed any other time aside from when you collected sample?
d. 114-116 can you explain where these abiotic measurements were taken? Water column ( top middle bottom), or in the benthic substrate? Do you think sampling location would change your results ( i.e. do you believe there is any stratification in your tanks? Why/why not?)
e. line 123-124, do you also use the integrated sampled to collect the bacteria samples?

Validity of the findings

1. I think most of these findings are valid, although I disagrees with the conclusion that richness is a positive relationship with co2 production, to me it looks unimodal across salinity suggesting a salinity optima. The authors could reaffirm their claim but explain how they used different models and their model was the best fit, or they should discuss how at intermediate salinities it appears CO2 is greatest, and the potential mechanisms for this finding + how it fits into the larger field of richness/function analysis in microbial/ ecosystem ecology ( maybe relate to species interactions (+,-) or intermediate disturbance hypothesis)
2. Data appear robust and replicated, no reason that I see to question their validity.
3. 336-337 I think I understand what the authors are trying to say about HGT, but If they want to talk about HTG it should be more than one sentence. This could include but is not limited to what genes increase survivorship under increased osmotic stress, or other studies that have investigated the transfer of osmoregulation genes. Also – I think the authors have the potential to use their 16s sequencing to see if any taxa/otu in their experiment are more associated with salt system, or are potential halophiles.
4. I think the conclusions could be greatly improved by (1) making them explicitly related to coastal ponds (2) concretely synthesizing and contextualizing their overall results in their final paragraph 359-365. Lastly, I would suggest they not end their paper on an intext citation. It would strengthen this paragraphs to being with that sentence ( we are answering the call to study taxonomic groups and functions across disturbance events)

Additional comments

Why are there no DO, NH4, PH data presented? If you aren’t going to show data then don’t talk about it in methods.
Present zoo plankton community data (abundance, diversity), you say that zooplankton functional groups are understood at the functional level ( lucky! I wish I could say the same for bacteria), so I think this should be a part of this paper. As is, you do not discuss this at all. Even if there is no change in functional groups of zooplankton that is still a very interesting finding.

Disagree with increased richness increases co2 production. While I agree it looks non linear, to me it appears that you have highest rates of co2 production at intermediate levels of microbial richness. I think this should be a much stronger component of this paper as it is a very interesting results. The authors should look for other papers which find a similar result to support their findings, or explain how their system is unique enough to present a different relationship with richness and co2 production.

I think you might want to reframe how you talk about CO2 production, as you don’t measure production over the course of the experiment, you only measure if at the end. Can you justify why this is appropriate? If I was the authors, I would make the argument that looking at the PCoAs ( figure 4 + 5) – the communities remain essentially unchanged throughout the experiment, and thus the communities at the final point are probably pretty representative of all sampling communities

Source tanks (a) – one think to consider is that by using these source tanks, you will like have selective for non-natural communities that are likely much less diverse than the true “ over-wash” communities. I understand that logistically traveling to collect samples from coastal ponds before each treatment day might not have been possible, but can you give any sort of details on how your “source” communities might relate to the natural communities? Do you have any community metrics for the natural communities? Maybe you could make the argument that while less diverse, the “source” communities were still comprised of the same dominant members and thus we feel the response of this source community is pretty representative of natural communities?

Source tanks (b) – do you have any metric of communities found in the source tank? Curious if they would match the treatment tanks or if they would separate on an ordination.

Terminology – The authors use the word dispersal twice in the abstract, setting the reader up to think this will be a paper about dispersal. However, in the actual text of the manuscript it only appears once. The research addressed, and the literature cited by the authors, indicate this is a paper that focuses on salinity effects on community structure and function rather than community member dispersal mechanisms.

Decomposition - I think your decomp results are some of your most interesting results and should be discussed more. There is a large debate in aquatic systems if decomp is faster at salt sites than fresh sites, and I think these results could contribute to that conversation. I also found it interesting that the mixed community was better at decomposing spartina than the salt community. If you can do C:N on the litter used in this study that would be very interesting. If that is not possible, the three plant species you chose are very commonly used and described, I am sure you could pull their C:N values from some papers. That might be interesting to think about when interpreting these results.

Reviewer 2 ·

Basic reporting

Overall, the manuscript is written with clear and unambiguous language. The study is on a topic broadly interesting to many people in various fields and the general PeerJ readership. I believe with some major revisions, this study can be published in PeerJ. However, as written, the manuscript lacks many of the appropriate literature references and background needed to give the readers appropriate context. I have detailed in this in the "general comments to the authors" section.

Experimental design

The general experimental design and research question is sufficient. The authors should revise the mesocosm descriptions to be more clear. I have detailed this and other edits in the "general comments to the authors" section.

Validity of the findings

I have some concerns in regards to this section. Overall, the data is interesting, however, the authors could expand upon the results they have with data they have at hand. This manuscript would benefit from an increased analysis of the OTUs present and how they correlated to the factors measured beyond salinity. Do the authors see any changes in abundances of an OTU or OTUs that relate to changes in carbon mineralization at the mid species richness? Were those isolates native or brought in with mixing? Or, if salinity negatively impacted thse functions what OTU changes could have influenced that? What insights can be garnered from those OTUs in their potential function? While richness can tell you about the diversity in the community it doesn't account for potential fluctuations in an organism or organisms that may relate to the loss of an ecosystem function. Further, while richness can increase, it may not be the immigration of organisms but rather the change in native taxa that resulted in the correlation. Conversely, increased richness may result in communities that are more resilient because of the functional redundancy provided. I think more detailed analyses of this data placed in context with other work on community resilience and salinity impacts could help shed some light on this and add to the overall manuscript.

Additional comments

Overall, the manuscript is well written and uses unambiguous language. A study on how salinity impacts ecosystem function and community composition is very interesting to broad fields and to PeerJ readers. However, the manuscript needs major revisions (discussed below) before it is ready to be published. The authors should focus on adding more context to the introduction, clearing up the mesocosm setup in the methods, and adding more details on OTU abundance and their potential relationship to the measured ecosystem functions. I want to stress I believe this manuscript is publishable and the data is interesting. I believe more context in the introduction with a better set up to the hypotheses and questions being tested and asked, respectively, will help considerably.

Introduction

The introduction is lacking a lot of literature, a proper review of the current knowledge, and many details on how salinity may impact ecosystem functions in aquatic systems. As written, the introduction felt more of a review of the study and methods done in it. The only introduction to the current field is lines 29-32 and paragraph two (lines 38-48). This made it hard to connect the importance of this study to what was being argued in the discussion and, most importantly, why this study was done in the first place. Moreover, the introduction would benefit from the addition of literature related to zooplankton/bacteria communities and salinity. The only literature in the introduction discussing aquatic communities of zooplankton or bacteria was Paver et al. (2018), an interesting study but was focused on the evolutionary transitions rather than the impact of salinity on community composition and ecological function. The rest of the studies focused on plants, insects, salmoids, and amphibians. A few pieces of literature that could help shape this introduction to focus more on the organisms being studied are:
-Langenheder, S., Kisand, V., Wikner, J., & Tranvik, L. J. (2003). Salinity as a structuring factor for the composition and performance of bacterioplankton degrading riverine DOC. FEMS Microbiology Ecology, 45(2), 189-202.
-Dupont, Chris L., John Larsson, Shibu Yooseph, Karolina Ininbergs, Johannes Goll, Johannes Asplund-Samuelsson, John P. McCrow et al. "Functional tradeoffs underpin salinity-driven divergence in microbial community composition." PloS one 9, no. 2 (2014): e89549.
-Lozupone, Catherine A., and Rob Knight. "Global patterns in bacterial diversity." Proceedings of the National Academy of Sciences 104.27 (2007): 11436-11440.
-Eiler, A. et al. Productivity and salinity structuring of the microplankton revealed by comparative freshwater metagenomics. Environ. Microbiol. 16, 2682–2698 (2014).
-Logares, R. et al. Infrequent marine-freshwater transitions in the microbial world. Trends in Microbiology 17, 414–422 (2009)

The introduction must set up the reader understand the problem that was being addressed, what other researchers have learned from their research into the problem, and how this manuscript is going to compliment and add new data to the previous research. Some topics I would like to see discussed are: 1) how are zooplankton and bacteria communities broadly influenced by salinity changes (e.g. fluxes in richness or shifts in structure) or other factors, 2) how do the change to (1) influence the functional genes present (e.g. genes related to decomposition or other important cycles) or ecological processes (e.g. nutrient cycling) occurring in these systems, 3) What has been missing and how does this study complement or improve upon that.

Specifics:
Lines 30-32: None of the citations used in these are examples of zooplankton or bacteria being impacted. While it is helpful to show how other communities are impacted, I am not sure how insects or angiosperms studied in Gutierrez-Canovas et al. and Zhang et al., respectively, give a good indication of how zooplankton or bacteria are impacted by salinity. Further, Mayfield et al. does not discussion salinity at all and is not appropriate to be used in this case.
Lines 34-36: This should be moved to the last paragraph as an introduction to what you will discuss later on and why you did this study. In this spot, it is confusing since there has not been much discussion about any of the important literature.
Lines 37-48: I think this is a good start to the introduction and should be combined with lines 30-32 and then greatly expanded upon. Limit or add more literature review of how the organisms being examined in this study (zooplankton and bacteria) are impacted by salinity changes or intrusions. A few studies to examine can be found referenced above.
Lines 56-58: Salinity is also an important determinant of bacterial communities as well. This should be emphasized.
Line 58: Stagg et al. is not an appropriate reference. The focus is on soil environments and on plant communities and does not measure bacteria richness at all.
Lines 62-75: The authors would be better served by not focusing on a review of the study and what you did. Shorten this down to two-three lines of a review of the methods, the take-home results, and why this is interesting data.

Methods

The general overview of the methods I understand; however, I found the specifics on how the mesocosms were made and maintained a bit confusing. I would like to see that section of the methods cleared up. See below for the details on what was confusing.

Specifics:

Lines 76-84: How were these mesocosms put together? What water was used to fill the experimental ponds? Was that water sterile or from the tap? If it was tap water, could that community present (usually as a biofilm) influence the microbial community you were examining?
Lines 81-82: How were the five different source water used? As written, I am assuming that initially all five were dumped into each tank; however, it is unclear if that was the case. If each treatment was seeded with a different pond how might that influence how the communities respond over the duration of the experiment? For instance, if you seeded one with a starting community that was at a salinity at 5 then it most likely all ready had a starting community more resistant to salinity fluctuations (fresh to brackish) vs. a community you seeded from a pond at a salinity of 0. This is important since you are comparing all the different treatments as if you began with a similar starting community. Further, for the source tanks, where were these communities from?
Line 81: The use of bacterioplankton is incorrect here and throughout the manuscript. Since the water was not filtered to remove particle-associated organisms this study is looking at whole bacterial communities rather than only the plankton portion.
Lines 133: delete “concentrated from” and replace with “extracted from”.
Lines 137-138: redundant to the line above.
Line 139: Since the water sample was not size-fractionated, bacterioplankton were not focused on. The water samples most likely represent both particle-associated and free-living organisms.
Lines 139-141: What chemistry was used?
Lines 142: The raw sequences would contain both archaea and bacteria unless there was a pre-step filtering process. If a filtering process was done prior to analysis it would need to be stated. Otherwise, the sentence should be corrected to “raw sequences”.
Line 144: Silva database should be cited
Line 146: Terminology: Sequences aren’t binned, a term often used in metagenomics, but most likely clustered. If they were clustered in MOTHUR which program was used (e.g. cluster_split() or?).
Lines 161-162: How was “and the interactions between time and salinity and salinity and mixing” incorporated into the model? Further, can the authors expand how “… random effect of replicate over time to account for repeated measure” was calculated and incorporated? It may be helpful by putting this all in a equation form.
Lines 178-179: Since you are using these two as proxies, it would be helpful to describe why in the introduction? Why use these two over primary production or other nutrient cycles.


Results

Lines 229-238: There is a ton of noise in this data between and within treatments. For instance, by eye, it would seem that the decrease in richness was largely driven by the increase in mescosm salinity 0 and decrease in the mesocosm salinity 9. The other two mesocosms (salinity 5 and 13) would seem to have almost no change on average. I would be curious if there was a statistical difference between mesocosm salinity 0 and mesocosm salinity 13 at day 45 or a statistical difference between salinity 5 or salinity 13 at day 0 and day 45, respectively. For instance, if you follow salinity 5 over time it would appear to increase in richness compared to day 18, but be more similar to day 0. Moreover, while there is a lot of fluctuation in richness for salinity 13, it seems it stayed pretty consistent with the exception of one point that saw an extreme drop in richness. The only system that appears to have a dramatic decrease in richness is mesocosm salinity 9. For that reason, I think it would be important to analyze these different mesocosms individually to see how the community and richness change over time rather than all together. The individual trends could then be used to help facilitate downstream analyses for comparing how salinity impacts.
Figure 3: Why on day 45 do the dots appear to shift over from their designated salinities? Did the mesocosms overall salinity?

Section 0.5.2: For the PCoA done, how was this created? What distance was used? Since PCoAs don’t take abundance counts, did the authors use UniFrac to calculate distance matrixes? If so, how was this done (e.g. MOTHUR)?

Section 0.6.2: The general trend is to increase, however, a look at the data would show that mid-level richness there were massive spikes in carbon remineralization that then goes back down. Why do the authors believe this happened? Are there any OTUs that the authors can tie to these spikes (e.g. specifically correlated)? Why are the authors only using richness and not the OTUs? Like indicator species, correlations of OTUs to the environmental data collect (e.g. carbon remineralization or decomposition) would massively improve this manuscript. It would help put into context what organisms were involved in it? Were the organisms that were not really abundant and then spiked? Were they organisms that potentially weren’t there but then introduced through mixing? These type of questions help put context to the increase, decrease or no change in richness since abundance is not considered in richness calculations. For instance, over time richness may not change, but the OTUs present may fluctuate. Therefore, the authors may miss relationships between fluctuations of an organism and decrease/increase in decomposition or remineralization.

Discussion
Much of the literature used in the discussion is on zooplankton or higher-level organisms, yet the majority of correlations and trends were found with bacteria. The literature should reflect that to help put your positive results into context of what has previously been found with bacteria and abiotic changes and their impact on ecosystem function.
Line 301: This hypothesis was not introduced anywhere else in the manuscript. If this is something you want to discuss it should be at the end of the introduction so that we know why you are doing the methods you are.
Lines 324-325: Is it the change in richness (e.g. more OTUs) or the increase in abundance of an individual OTU or OTUs that led to this correlation. By looking at abundances increase richness would mean the importance of the introduction of new OTUs from other sources (e.g. immigration) rather than the local community. If the latter is the case then this should be discussed.
Lines 333-335: If this is the case then how would you get an increase in richness? Increased richness is the result of increased immigration from outside sources. Therefore, if mixing was not where the increased richness was from, where were the new OTUs from (the moss?).
Lines 340-341: Stagg et al. did not measure bacterial richness. They inferred some about salinity impacts on microbial community structure and decomposition but they provide no direct data of richness and decomposition as suggested by these lines.

---

## Round 0.2 · Major Revisions

Dear authors,
Thank you for the revised version and the effort to improve the previous version of the ms. Despite the positive comments of one of the referee, I I still have important concerns regarding the new changes and invite you to make a further revision. Authors have included lot of text and it need now new revision as completely new information have been added, representing an important part of the ms. Authors should pay attention in reviewing the entire text of the ms to ccount for a correct article style (sometimes I have the impression to be reading a report rather than an article, including figure and table legends), some English corrections and verb time inconsistences (mixing past with present tense). Please use other underline in the next version; it is rather uncomfortable to read the text with this underline style! Thanks!
Specific points are detailed below:
- Paragraph including lines 66-86 (effect of salinity on bacterial and zooplankton communities): information must be synthesized. In the current form it seems more a review. It is not necessary to provide all these examples. Just explain the most relevant and cite the rest.
- Paragraph including lines 87-90 (on coastal wetlands): you can delete it.
- Paragraph including lines 90- 109: as previously mentioned, information must be synthesized, keeping the most relevant cases.
- Lines 113-125 (on carbon cycle): It also needs reduction, by focusing in the most important information.
-Line 127: “environmental change” is too vague. Be more precise or delete it.
- The aim of the study and hypothesis must be restructured and clearly stated. I suggest putting them in the last paragraph of the introduction section. Currently it is rather disperse (e.g. lines 109-102, lines 123-125, lines 126-142). Also remove the results from the introduction section (Lines 144-149).
- Lines 126 and 129: redundant “specifically”
- Line 152: replace “good place” by “suitable place”
- Line 153: vulnerable to changes is too vague, please be more specific
- Line 185: “peat moss and sand were purchased”. This information is not important; it is better to describe it, if it is something special, or remove it. It is not common to put this information in an article.
- I’m not native English speakers, but I think that English should be reviewed throughout the text; for example: Line 197: “We don’t except our tank to (BE) stratified”
Line 390: Poly(salinity 2): ??
Lines 424-451: most of this paragraph is not suitable for a discussion section but for a result section. Here you must DISCUS the most relevant results on the basis of main hypothesis of the study.
481-484: The home-field advantage hypothesis is not well presented here. You must start discussing your results on the basis of this hypothesis and after that, to compare with other studies. I find it a little bit odd the way it is introduced.
Line 494-498: mixing verb tense (past and present); use past tense.
Tables and figure legends should be rewritten to read as standard figure and table legends of articles

·

Basic reporting

N/A

Experimental design

N/A

Validity of the findings

N/A

Additional comments

The authors answered all of my questions and concerns from the first review. I believe this has greatly increased the focus and reproducibility of the experiment. I have no further revisions for this manuscript. My only suggestion to the authors would be a potential rephrasing of their 3rd intro paragraph (Lines 53-65). I completely understand the authors are trying to unpack the effects salinity has on biological communities. However, the claims about microbial communities could be adjusted. For example they end the paragraph by saying there will be a decrease in bacterial functional processes with increasing salinity. While some processes might be suppressed (e.g. methanogenesis, nitrification), other processes would be enriched for ( e.g. sulfur oxidation and reduction). Therefore, I would suggest the authors adjust this paragraph to say that salinity is known a known driver of microbial communities and the biochemical cycles they mediate. This claim is widely accepted, whereas I would not be surprised to see papers that find moderate increases in salinity increase the abundance/functional potential of certain freshwater communities. However, this is only a recommendation, and I do not this it is a required edit.

---

## Round 0.3 · Minor Revisions

Dear authors,

Thank you for this new revised version of your manuscript. It has been significantly improved and now I’m satisfied with this. I have only minor comments, most of them concerning figure captions. As I suggested in my previous letter, figure captions should provide enough information that a reader can understand the data presented without referring to the text, but NOT explaining the results. Maybe I was not sufficiently concise. Figure caption should contain a concise description of the graphic allowing a reader to understand the figure. You can start with a short title (an overall descriptive statement of the figure) followed by additional text explaining panels, error bars, symbols, etc. But don’t provide explanations of results (e.g. “We do not observe separation by…”, “bacterial richness increased with salinity…”, etc). Explanation of results showed in the figure should be placed exclusively in the results section, but not in the figure captions. Specific comments are raised below. Authors should take into account these points before final acceptance of the ms.
Line 339-340: “an salt gradient”  correct English to “a salt gradient”
Line 447: Acceleratiextremeng  correct
Figure 1 should start with something like “Experimental design showing the four salinity treatments and the two dispersal treatments” instead of “To test the effect of species mixing across a salinity gradient we created…:”
Figure 2 should start with something like “PCoA for the relationship between salinity and zooplankton community”. Please, remove the explanation of results: “Freshwater communities are consistently distinct from other communities over time”…
Figure 3 should start with something like: “PCoA for the relationship between salinity and bacterial communities”. Please remove explanation of results: “Observed bacterial communities segregate into two [..97 ] clear groups along PCoA 1,freshwater and salt communities. The saline communities additionally separate along the second Axis. We do not observe separation by dispersal treatment”.
Figure 4: you should not start with this sentence “We are unable to determine how zooplankton richness changes across salinities…” Instead, describe what this figure represents.
Figure 5: Again, don’t describe the results here (“Observed bacterial richness increased as salinity increased but this effect lessened over time and reversed by the final time point”). Instead describe what the figure represents. For example, something like: “Variation in microbial richness in response to salinity treatments”
Follow the same suggestion for figures 6, 7 and 8.
Figure captions should be reordered (in the revised version it follows as 1, 4, 5, 2, 3, 6, 7, 8)

---

## Round 0.4 · accepted · Accept

Dear authors, thank you for the new version of the manuscript; all my comments have been addressed and accordingly I recommend its publication. Congratulations!