# Supplementary material for: Effects of diversity and coalescence of species assemblages on ecosystem function at the margins of an environmental shift
Source: PeerJ. 2020 Mar 9;8:e8608. doi: 10.7717/peerj.8608 (PMC7067187; doi:10.7717/peerj.8608)
Supplement: Supplemental Information 1 [file peerj-08-8608-s001.pdf]

9 SUPPLEMENT

9.1 Coastal Ponds

Coastal ponds were sampled in the inner and outer banks of North Carolina on May 3, 2015 in order to collect organisms with which to seed our experimental ponds.

Table S1. The environmental parameters of the coastal ponds where we collected zooplankton and bacterialplankton for our experimental tanks

GPS	Temp (°C)	Salinity (PSU)	Ammonium (mg/L)	pH
35°49.207 N 75°33.755	28.9	10.94	5.94	8.65
35°36.6321 N 75°28.074	28.8	5.26	2.61	9.37
35°41.89 N 75°29.141	22.9	15.65	8.73	8.14
35°49.232 N 75°34.004	35.5	0.17	0.14	6.34

9.2 Model Coefficients

Table S2. Coefficients for zooplankton family richness and observed bacterial richness models. Parameter estimates and standard deviations are on the log scale.

Model	Parameter	Estimate	SD	*z*	*p*
Zooplankton Richness	Salinity	-0.02	-0.01	-1.5	0.115
	Dispersal	0.09	0.11	0.87	0.38
	Time	-0.002	0.003	-0.72	0.47
	Salinity:Dispersal	-0.06	0.014	-0.45	0.62
	Salinity:Time	0.0001	0.0004	0.29	0.76
Bacterial Richness	Salinity	0.035	0.008	4	4.97e-05
	Dispersal	0.14	0.1	1.4	0.15
	Time	0.008	0.002	4.07	4.51e-05
	Salinity:Dispersal	-0.02	0.01	-1.8	0.06
	Salinity:Time	-0.001	0.0003	-4.2	2.33e-05

9.3 Source Tank Alpha Diversity

9.3.1 Zooplankton Richness

Zooplankton richness in source tanks decreased across salinity (estimate(log scale)=-0.02, standard error(log scale)=0.011,t = -2.1, p = 0.04) (Figure S1. We are unable to detect the main effect of day (estimate(log scale)=-0.006, standard error(log scale)= 0.003,t = -1.7, p =0.09). We were also unable to parse the affect of the interaction between time and salinity (estimate(log scale)=-0.0002, standard error(log scale)= 0.00049,t =-0.4 , p = 0.66).

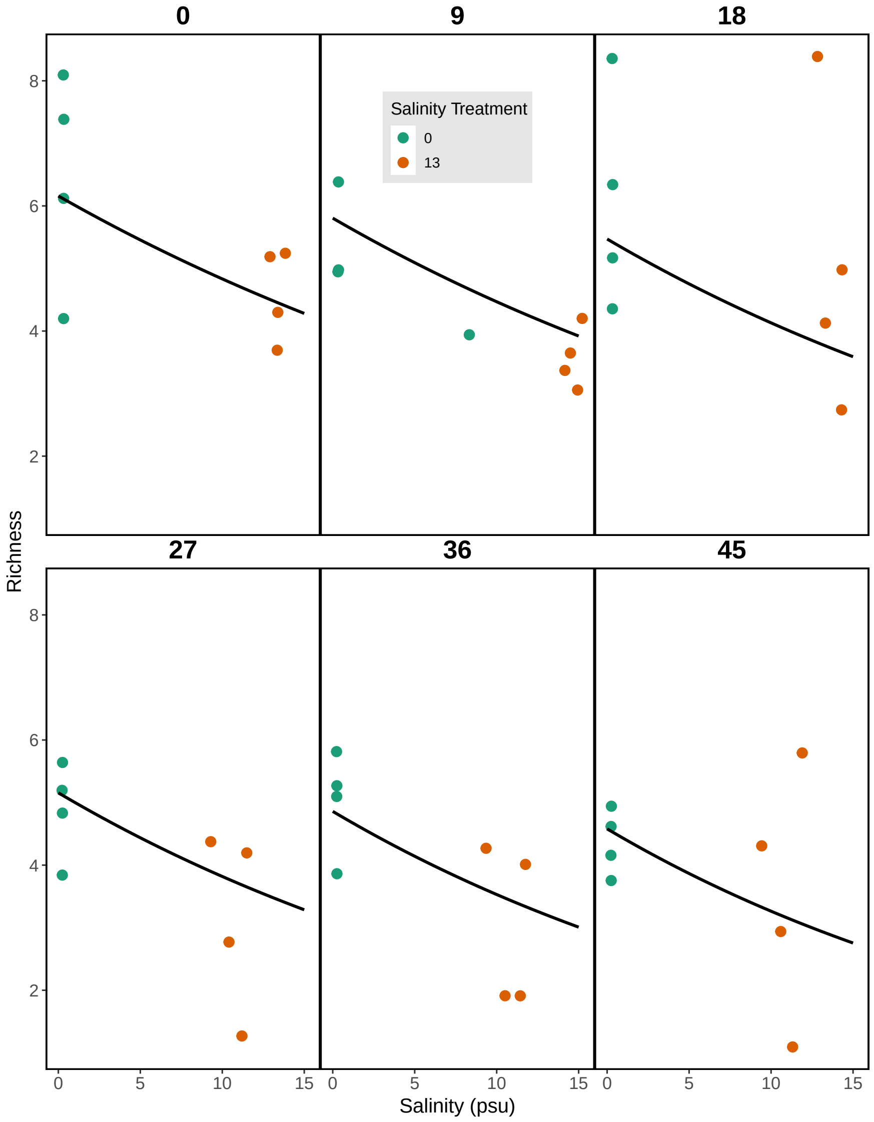

Figure S1. Each Panel represents a single sampling day. Point colors represent salinity treatment. Lines are predictions lines from our model.

9.3.2 Bacterial Richness

We are unable to distinguish bacterial source tank richness by salinity (estimate(log scale)=0.014, standard error(log scale)=0.14,z=1.003, p=0.3), day (estimate(log scale)=-0.0009, standard error(log scale)=0.004,z=-0.203, p=0.8) or the interaction of salinity and day (estimate(log scale)=-0.0005, standard error(log scale)=0.0005,z=-0.96,p=0.3).

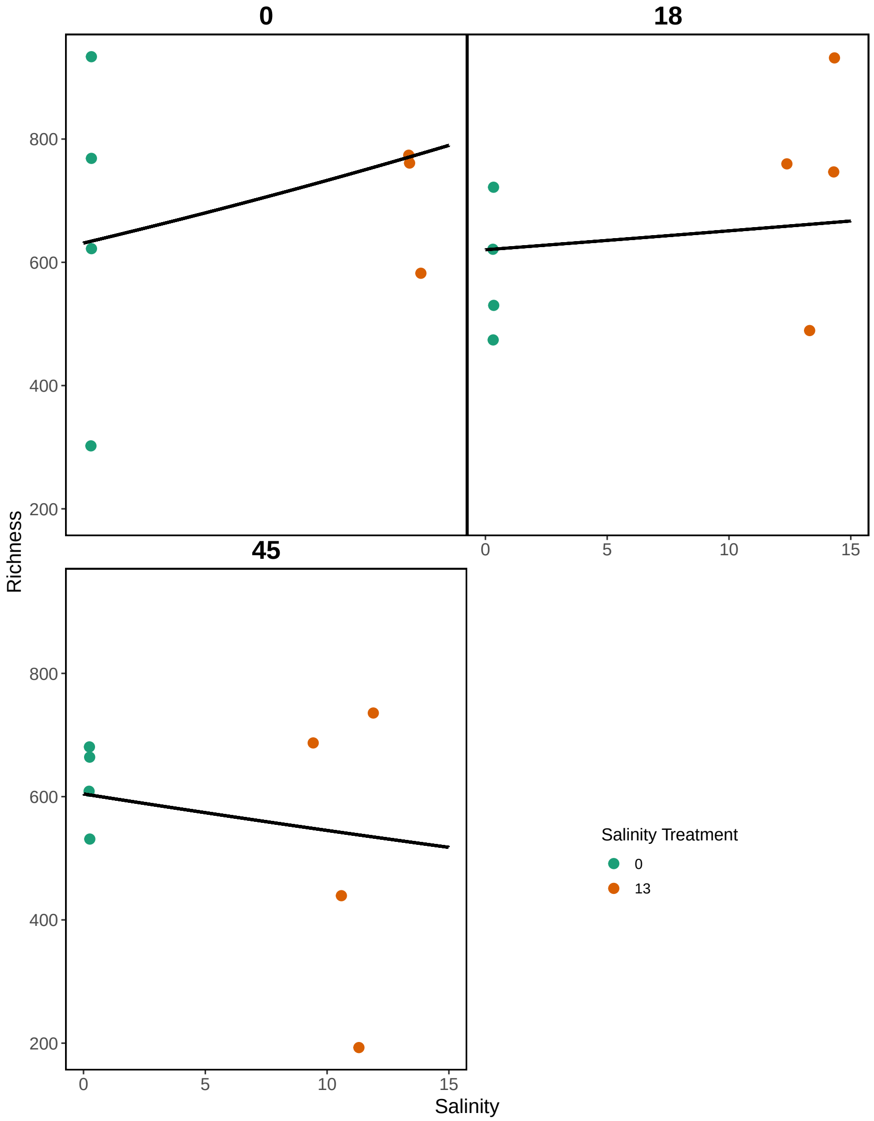

Figure S2. Each Panel represents bacterial richness at a single sampling day. Point colors represent salinity treatment. Lines are predictions lines from our model.

9.3.3 Shannon Diversity Index

We are unable to detect the affect of salinity, time or their interaction on Shannon Diversity of zooplankton communities (all $p > 0.05$).

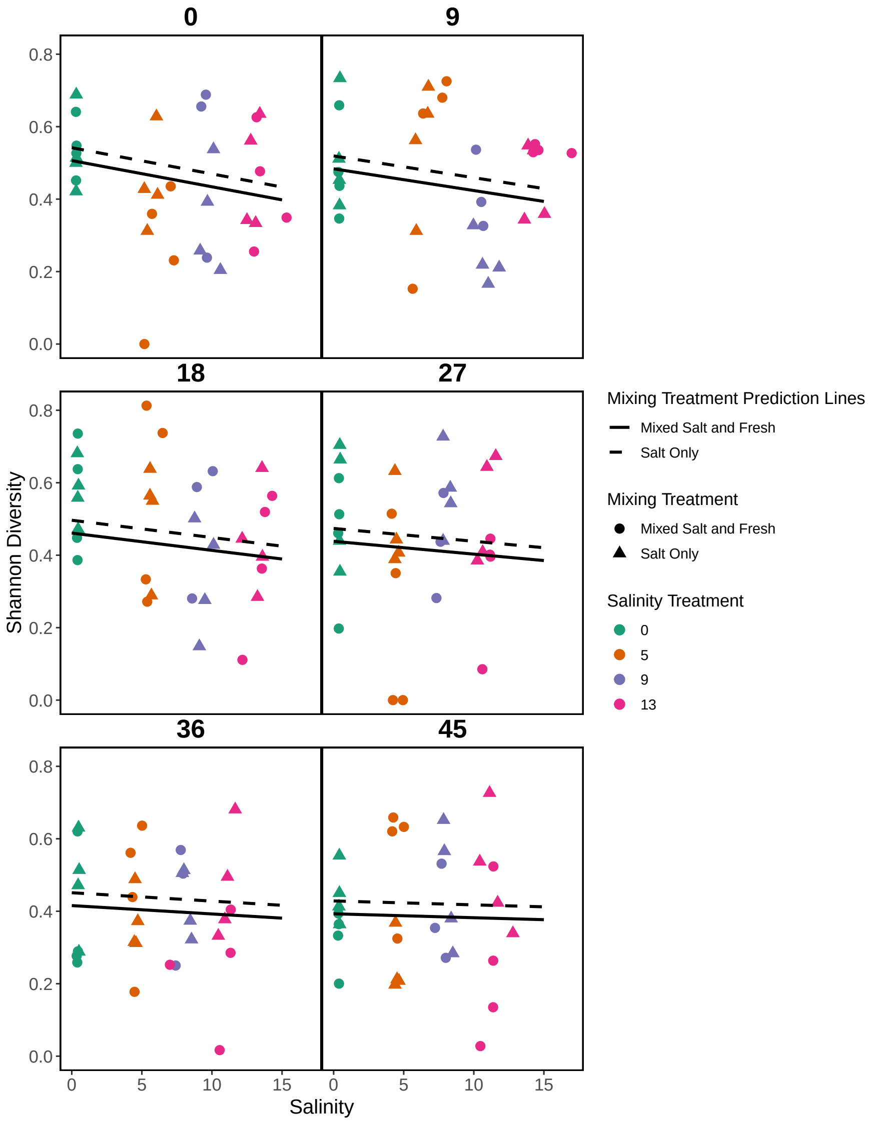

Figure S3. Each panel is sampling day. Colors represent salinity treatment. Shapes represent mixing treatment. Lines are model predictions.

For bacterioplankton communities we find that Shannon Diversity increases across as salinity increases (Estimate= 0.055, standard error = 0.02, p=0.008) and over time (Estimate = 0.016, standard error = 0.006, p = 0.008). The positive increase across salinity is decreased over time (estimate = -0.002, standard error = 0.0008, p = 0.004). We are unable to detect an affect of dispersal treatment ($p > 0.05$).

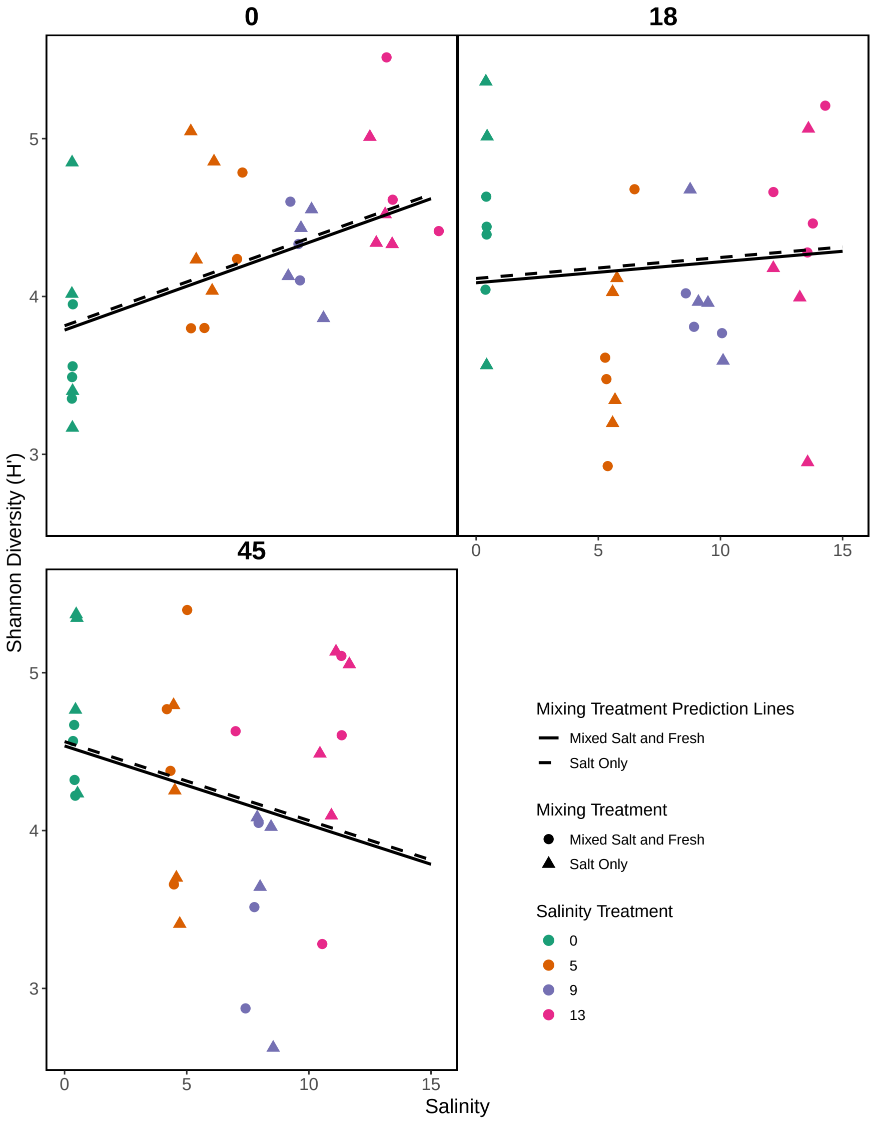

Figure S4. Each Panel represents bacterial shannon diversity at a single sampling day. Point colors represent salinity treatment. Lines are predictions lines from our model.

9.4 Beta Diversity

9.4.1 Zooplankton

Zooplankton communities in source tanks were separated clearly by salinity along the primary axis, which explained 45% of the variation. The secondary axis explained 14% of the variation in communities but the communities are not clearly separated along this axis.

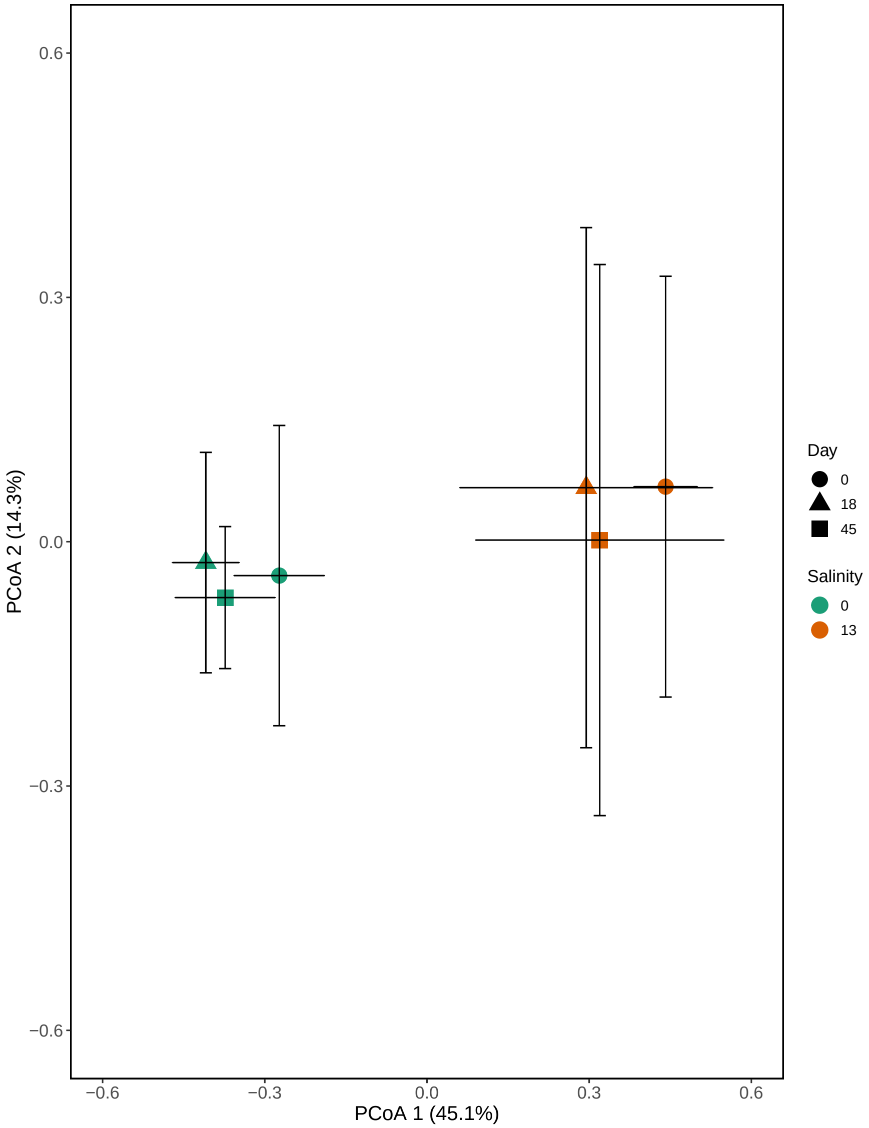

Figure S5. Points represent the centroid of the community structure. Error bars are standard deviation. The top panel is the starting structure, the middle panel is day 18 and the bottom panel is the final day.

Zooplankton source tank communities were very similar to other tanks in the same salinity in other treatments throughout the experiment.

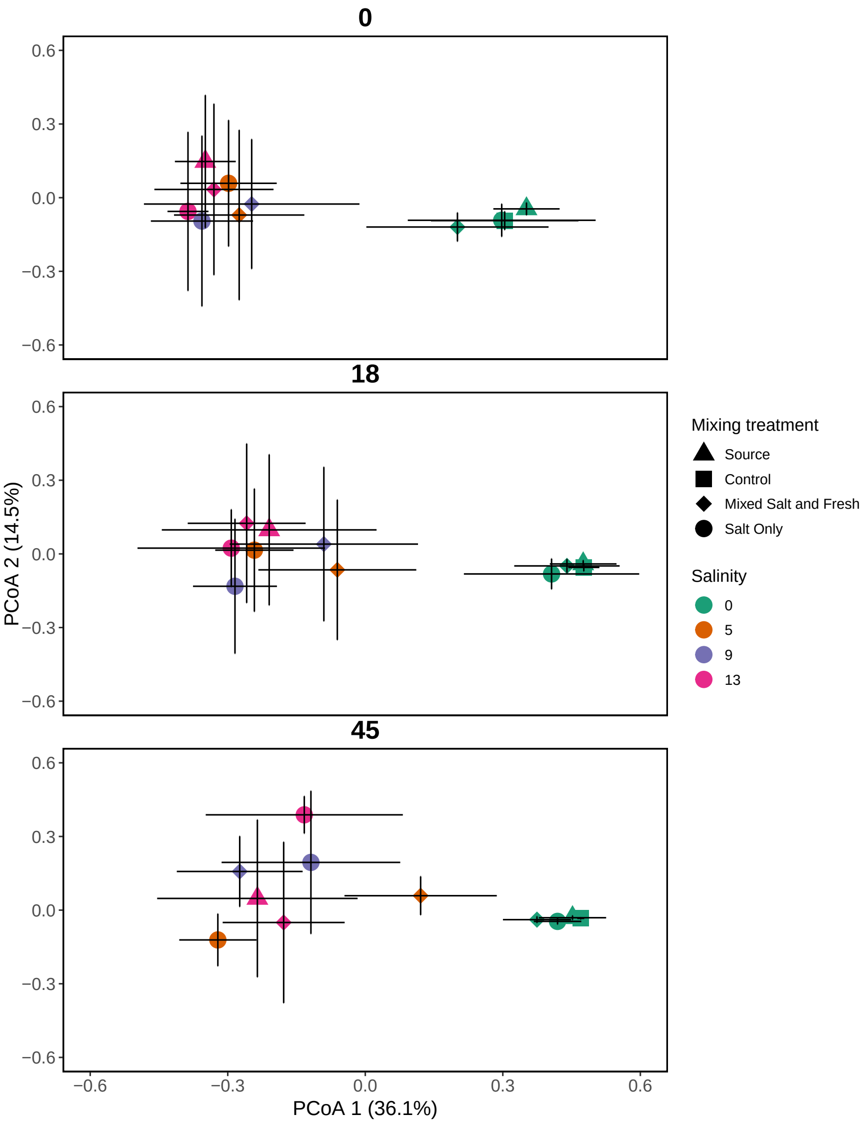

Figure S6. Points represent the centroid of the community structure. Error bars are standard deviation. The top panel is the starting structure, the middle panel is day 18 and the bottom panel is the final day.

9.4.2 Bacterial

For bacterial communities in the source tanks the primary axis explained 29.3% of variation, showed a clear distinction between the source fresh and source salt tank communities. The secondary axis, which explained 9.7% of the variation, showed separation of the communities by date; the freshwater source community showed day 0 and 18 the furthest apart, with the day 45 community more similar to day 0. For the saltwater source community, day 18 and 45 communities are furthest apart, with day 0 located in the middle (Figure S7.

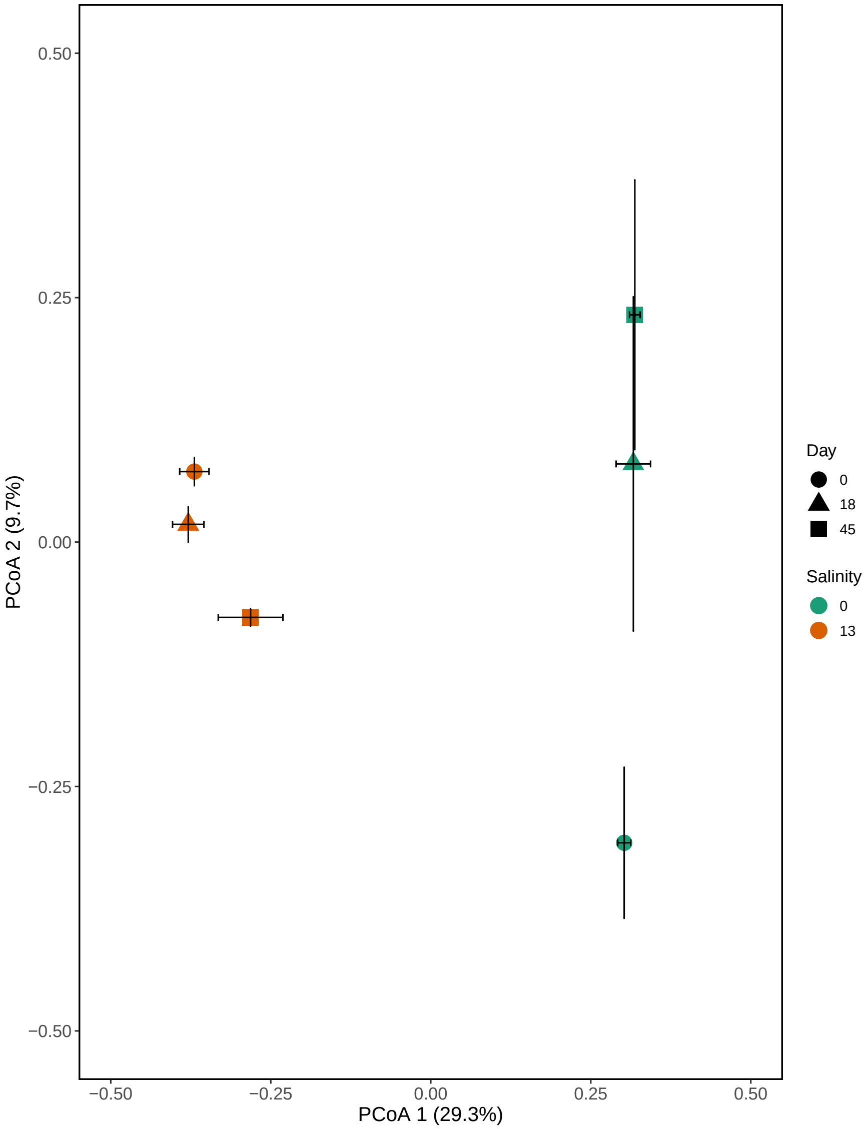

Figure S7. Points represent the centroid of the community structure. Error bars are standard deviation. The top panel is the starting structure, the middle panel is day 18 and the bottom panel is the final day.

Microbial source tank communities were very similar to other tanks in the same salinity in other treatments throughout the experiment.

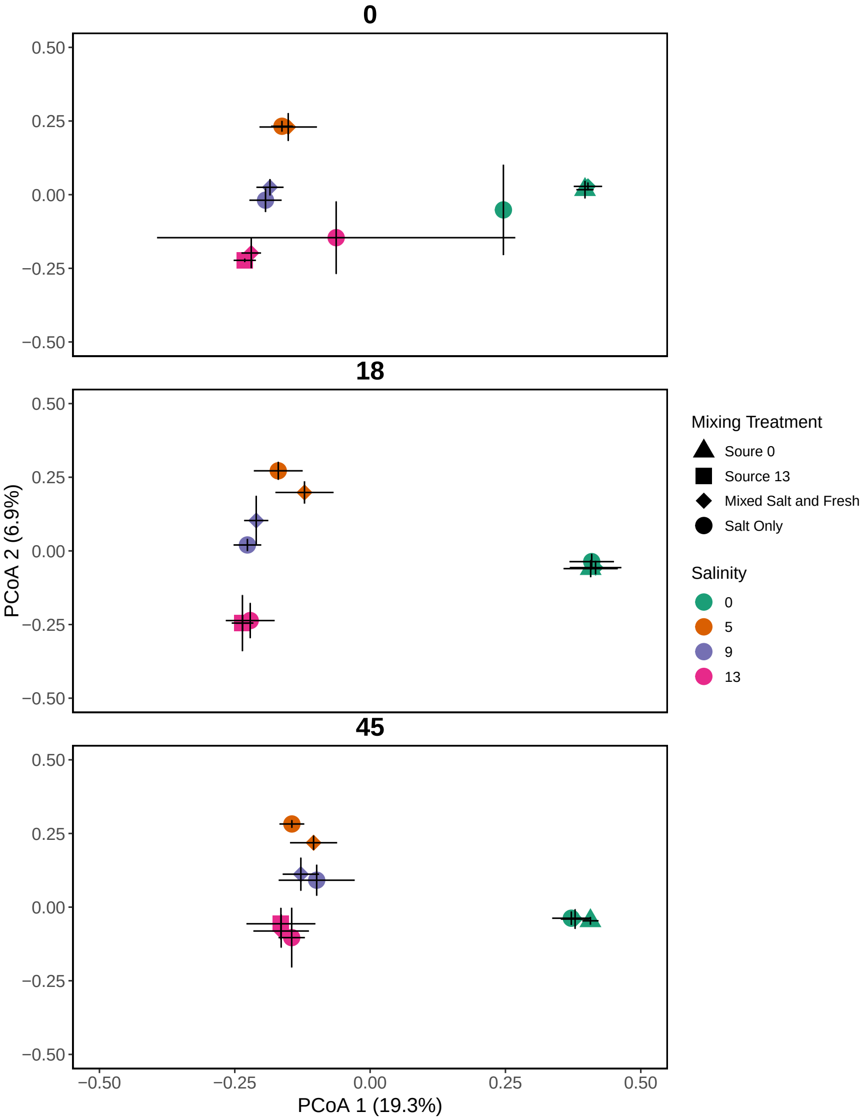

Figure S8. Points represent the centroid of the community structure. Error bars are standard deviation. The top panel is the starting structure, the middle panel is day 18 and the bottom panel is the final day.

9.5 Mesocosm Environment

The salinity in each tank was relatively constant across the duration of the experiment.

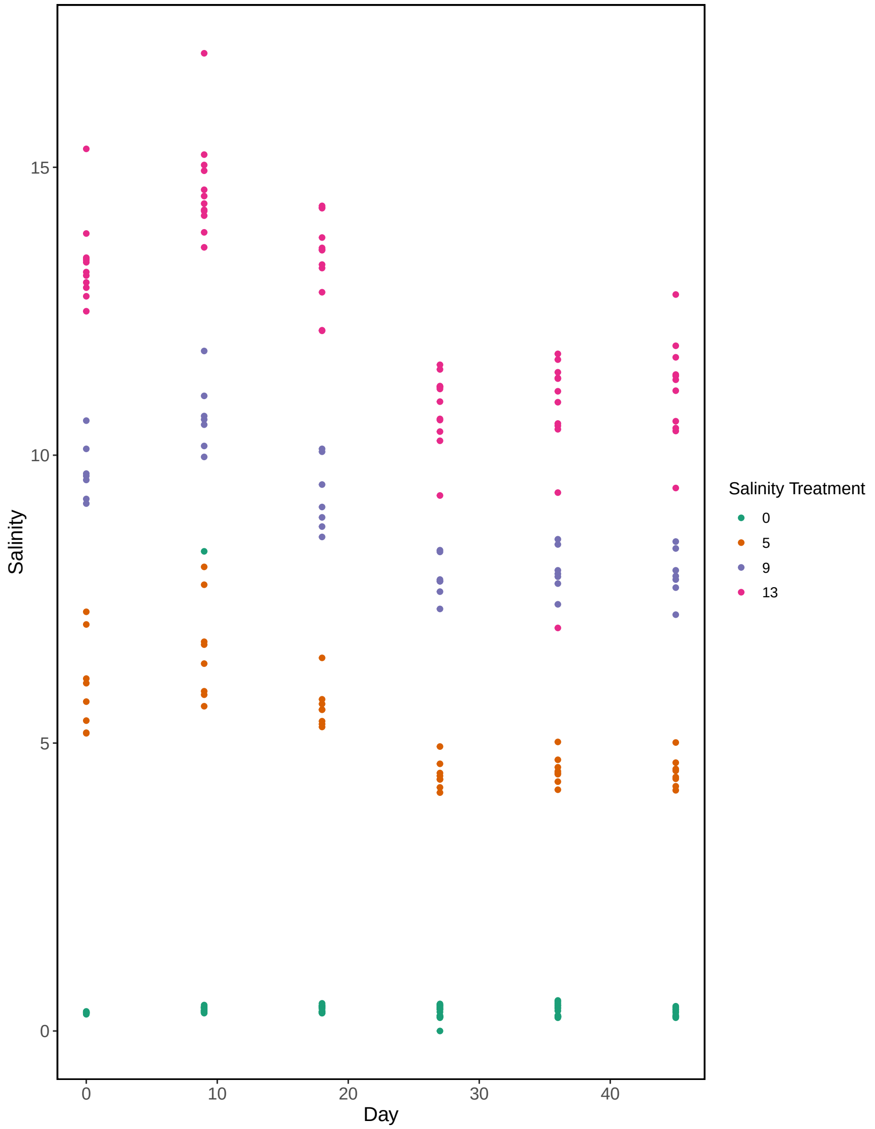

Figure S9. X-axis is each sampling day. Y-axis is measured salinity for each replicate. Colors represent the salinity treatment.

Table S3. **Bacterial Indicator Analysis:** Abbreviated species indicator analysis table (full table available at: https://github.com/PeraltaLab/CSI_Dispersal/. Table displays the OTUs with the highest indicator values (IndVal) for each Salinity treatment (cluster) (cluster 1 = salinity 0, 2 = salinity 5, 3 = salinity 9, 4 = salinity 13). The phylum, class, order, family, and genus are matched with each OTU.

OTU	Salinity	IndVal	Prob	Phylum/Class/Order/Family/Genus
Otu00150	0	0.9907	0.001	Proteobacteria/Proteobacteria_unclassified/Proteobacteria_unclassified/ Proteobacteria_unclassified/Proteobacteria_unclassified
Otu00045	0	0.9903	0.001	Proteobacteria/Alphaproteobacteria/Rhodospirillales/ Rhodospirillales_unclassified /Rhodospirillales_unclassified
Otu00009	0	0.9831	0.001	Proteobacteria/ Betaproteobacteria/ Burkholderiales/ Burkholderiaceae/ Polynucleobacter
Otu00105	0	0.9796	0.001	Verrucomicrobia/Spartobacteria/Spartobacteria_order_incertae_sedis/ Spartobacteria_family_incertae_sedis/ Spartobacteria_genera_incertae_sedis
Otu00134	5	0.9361	0.001	Proteobacteria/Betaproteobacteria/Betaproteobacteria_unclassified/ Betaproteobacteria_unclassified/ Betaproteobacteria_unclassified
Otu00039	5	0.8889	0.001	Bacteroidetes/Flavobacteria/Flavobacteriales/Flavobacteriaceae/Flavobacterium
Otu00089	5	0.8522	0.001	Proteobacteria/Betaproteobacteria/Burkholderiales/Alcaligenaceae/Alcaligenaceae _unclassified
Otu00052	5	0.8226	0.001	Bacteroidetes/Sphingobacteria/Sphingobacteriales/Cyclobacteriaceae/ Cyclobacteriaceae_unclassified
Otu00355	9	0.9449	0.001	Bacteria_unclassified/Bacteria_unclassified/Bacteria_unclassified/Bacteria_unclassified /Bacteria_unclassified
Otu00067	9	0.8253	0.001	Bacteria_unclassified/Bacteria_unclassified/Bacteria_unclassified/Bacteria_unclassified /Bacteria_unclassified
Otu00198	9	0.8044	0.001	Planctomycetes/Phycisphaerae/Phycisphaerales/Phycisphaeraceae/ Phycisphaera
Otu00016	9	0.7730	0.001	Bacteria_unclassified/Bacteria_unclassified/Bacteria_unclassified/Bacteria_unclassified /Bacteria_unclassified
Otu00324	13	0.9276	0.001	Proteobacteria/Alphaproteobacteria/Alphaproteobacteria _unclassified/Alphaproteobacteria_unclassified/ Alphaproteobacteria_unclassified
Otu00380	13	0.8686	0.001	Proteobacteria/Gammaproteobacteria/Alteromonadales/Alteromonadaceae/Haliea
Otu00111	13	0.8682	0.001	Bacteria_unclassified/Bacteria_unclassified/Bacteria_unclassified/Bacteria_unclassified /Bacteria_unclassified
Otu00227	13	0.8555	0.001	Bacteria_unclassified/Bacteria_unclassified/Bacteria_unclassified/Bacteria_unclassified /Bacteria_unclassified
